# Comorbidities in hidradenitis suppurativa: Role of metabolic syndrome and sexual dysfunction disorders in postoperative healing

**DOI:** 10.1016/j.jdin.2024.09.006

**Published:** 2024-10-26

**Authors:** Sowmya Ravi, Tatjana Mortell, Dylan Wolff, Amy Stein, Emily Coleman, Abigail Chaffin

**Affiliations:** a Tulane University School of Medicine, New Orleans, Louisiana; b Division of Plastic Surgery, Department of Surgery, Tulane University School of Medicine Tulane School of Medicine, New Orleans, Louisiana; c Pediatric Dermatology Research Alliance, Denver, Colorado; d Department of Dermatology, Tulane University School of Medicine, New Orleans, Louisiana

**Keywords:** clinical research, comorbidities, epidemiology, general dermatology, hidradenitis suppurativa, medical dermatology, plastic surgery, socioeconomic factors

*To the Editor:* Hidradenitis suppurativa (HS) is a chronic painful inflammatory folliculitis which has been associated with numerous co-morbidities such as obesity, metabolic syndrome, smoking, and autoimmune diseases in the literature.^1^^,^^2^ These comorbidities contribute to systemic inflammation and immunologic dysfunction that worsen the pathogenesis of the disease.^3^ In cases of severe disease (Hurley Stage III), surgical intervention including wide local excision is required for treatment.^1^

Given the documented association of co-morbid conditions, our study sought to better understand the impact of medical comorbidities on postoperative outcomes for patients with severe HS. We conducted a retrospective review of 65 Hurley Stage III HS patients (Table I) at a multicenter academic institution in New Orleans, Louisiana, from 2013 to 2023. Patients were identified from a single-surgeon panel utilizing CPT codes that covered excision of HS from numerous areas of the body and associated codes (10060, 10061, 11450, 11451, 11462, 11463, 11470, 11471, 15002, 15003, 15004, 15040, 17110, 17111). Recurrence was defined as recurrence of disease anywhere on the body after surgical excision.

**Table I:** Demographics and comorbidities

	Overall (*n* = 65)	Male (*n* = 15)	Female (*n* = 50)	*P*-value
Demographics				
Age; mean [range]	41.3 [16-70]	49.7 [25-70]	38.8 [16-67]	.023
Race				
Black	47 (72%)	10 (67%)	37 (74%)	.820
White	17 (26%)	5 (33%)	12 (24%)	.699
Hispanic ethnicity	3 (5%)	1 (7%)	2 (4%)	.999
Insurance provider				
Private insurance	47 (72%)	11 (73%)	36 (72%)	.260
Medicaid	7 (11%)	0 (0%)	7 (14%)	
Medicare	10 (15%)	4 (27%)	6 (12%)	
Self-pay	0 (0%)	0 (0%)	0 (0%)	
Unknown	1 (2%)	0 (0%)	1 (2%)	
BMI	34.9 (8.6)	35.1 (9.6)	8.3 (34.8)	.891
Smoking status				
Never smoker	41 (63%)	5 (36%)	36 (72%)	.044
Former smoker	10 (15%)	4 (29%)	6 (12%)	
Active smoker	13 (20%)	5 (36%)	8 (16%)	
Comorbidities				
Depression	35 (54%)	7 (47%)	28 (56%)	.733
Anxiety	30 (46%)	4 (27%)	26 (52%)	.153
Metabolic syndromes	14 (22%)	3 (20%)	11 (22%)	.999
Sexual dysfunction	23 (35%)	7 (47%)	16 (32%)	.463
Obesity	47 (72%)	10 (67%)	37 (74%)	.820
HIV	1 (2%)	0 (0%)	1 (2%)	.999
Iron deficiency anemia	12 (18%)	3 (20%)	9 (18%)	.999
Anemia of chronic disease	12 (18%)	2 (13%)	10 (20%)	.838
Malnutrition	4 (6%)	2 (13%)	2 (4%)	.480
IBD	15 (23%)	4 (27%)	11 (22%)	.979
HTN	43 (66%)	12 (80%)	31 (62%)	.327
HLD	30 (46%)	10 (67%)	20 (40%)	.128
DM	22 (34%)	4 (27%)	18 (36%)	.720
Rheumatoid arthritis	4 (6%)	1 (7%)	3 (6%)	.999
Psoriasis	4 (6%)	1 (7%)	3 (6%)	.999
Psoriatic arthritis	3 (5%)	1 (7%)	2 (4%)	.999
No associated disease	5 (8%)	0 (0%)	5 (10%)	.470
Number of comorbidities mean [range]	5.2 [0-12]	5.5 [0-10]	5.1 [1-12]	.645

*BMI*, Body mass index; *DM*, diabetes mellitus; *HLD*, hyperlipidemia; *HTN*, hypertension; *IBD*, inflammatory bowel disease.

The results of our study (Table II) indicate that patients with metabolic syndrome were 5.16 times more likely to have HS recurrence (*P* = .014), and patients with sexual dysfunction disorders were 5.84 times more likely to have HS recurrence (*P* = .002) relative to HS patients without these disorders. For each additional comorbidity, the odds of a recurrence increased by a factor of 1.48 (*P* = .029). Former smokers had an increased defect size by a factor of 3.62 compared to never smokers (*P* = .039), and for every additional medication, the defect size increased by a factor of 1.39 (*P* = .026).

**Table II:** Recurrence and defect size

	Recurrence		Defect size	
	OR (95% CI)	*P*-value	Exponentiated coefficient (95% CI)	*P*-value
Demographics				
Age	1.01 (0.98, 1.04)	.538	1.01 (0.98, 1.04)	.625
Race				
Black	2.28 (0.72, 8.07)	.174	0.50 (0.20, 1.23)	.129
White	0.50 (0.14, 1.57)	.250	1.95 (0.77, 4.92)	.153
Insurance provider				
Private insurance	[Reference]		[Reference]	
Medicaid	0.86 (0.15, 4.32)	.851	1.15 (0.29, 4.52)	.835
Medicare	0.29 (0.04, 1.30)	.138	1.27 (0.29, 5.55)	.749
BMI	1.03 (0.97, 1.09)	.378	1.05 (0.99, 1.10)	.056
Smoking status				
Never smoker	[Reference]		[Reference]	
Former smoker	2.03 (0.50, 9.02)	.326	3.62 (1.07, 12.23)	.039
Active smoker	0.41 (0.08, 1.57)	.218	0.89 (0.31, 2.57)	.827
Alcohol consumption >0	1.95 (0.60, 6.51)	.267	0.88 (0.33, 2.36)	.787
Comorbidities				
PCOS	0.94 (0.12, 6.12)	.952	2.60 (0.64, 10.58)	.177
Depression	1.29 (0.47, 3.60)	.619	0.72 (0.30, 1.72)	.448
Anxiety	1.71 (0.62, 3.79)	.299	0.46 (0.19, 1.09)	.076
Metabolic syndromes	5.16 (1.48, 21.23)	.014	1.92 (0.64, 5.80)	.239
Sexual dysfunction	5.84 (1.95, 19.15)	.002	1.26 (0.51, 3.12)	.610
Obesity	2.98 (0.90, 11.83)	.089	1.79 (0.64, 5.01)	.259
Iron deficiency anemia	0.66 (0.16, 2.38)	.537	1.88 (0.59, 5.98)	.280
Anemia of chronic disease	0.78 (0.19, 2.91)	.716	1.51 (0.31, 7.31)	.601
Malnutrition	1.46 (0.17, 12.84)	.715	6.55 (0.79, 54.16)	.080
Inflammatory bowel disease	2.30 (0.69, 8.01)	.177	1.86 (0.61, 5.61)	.266
HTN	0.51 (0.17, 1.46)	.208	0.64 (0.26, 1.61)	.338
HLD	1.31 (0.48, 3.63)	.597	0.93 (0.39, 2.26)	.878
DM	1.48 (0.51, 4.30)	.470	1.08 (0.39, 2.60)	.987
Rheumatoid arthritis	4.70 (0.56, 98.12)	.192	3.16 (0.54, 18.61)	.197
Other comorbidities	2.70 (0.60, 14.28)	.204	0.88 (0.37, 2.14)	.780
Number of comorbidities	1.48 (1.06, 2.17)	.029	1.10 (0.89, 1.35)	.360
Number of medications	1.38 (0.97, 2.02)	.081	1.39 (1.04, 1.85)	.026

*BMI*, Body mass index; *DM*, diabetes mellitus; *HLD*, hyperlipidemia; *HTN*, hypertension; *PCOS*, polycystic ovarian syndrome.

While prior studies have shown as association between metabolic syndrome and sexual dysfunction and HS, our study adds to the literature by characterizing the association of these comorbidities with disease recurrence in individuals with severe disease who have undergone wide-surgical excision.^4^^,^^5^ In addition, the association between HS and sexual dysfunction sheds light on the multifaceted nature of this debilitating condition and the impact on patients’ overall well-being and suggests that comorbid sexual dysfunction may exacerbate the underlying inflammatory process involved in HS pathogenesis leading to a higher likelihood disease recurrence.

It is critical to emphasize that while our findings highlight the challenges posed by comorbidities in HS, our data advocate for multidisciplinary preoperative assessment and management to optimize outcomes for patients with severe HS. We suggest patients with comorbid metabolic syndrome undergo medical optimization prior to surgery. We also suggest that disorder of sexual dysfunction be screened for during dermatologic and preoperative visits to further optimize patients prior to surgery to provide holistic care for HS patients.

In conclusion, metabolic syndrome and sexual dysfunction disorders were found to negatively impact postoperative healing in HS. An awareness of at-risk comorbidities and interprofessional management of these patients are essential for physicians caring for Hurley Stage III HS patients.

## Conflicts of interest

None disclosed.
